# *“If I have only two children and they die… who will take care of me?”* –a qualitative study exploring knowledge, attitudes and practices about family planning among Mozambican female and male adults

**DOI:** 10.1186/s12905-017-0419-6

**Published:** 2017-08-22

**Authors:** Rehana Capurchande, Gily Coene, Kristien Roelens, Herman Meulemans

**Affiliations:** 1 Department of Sociology, Eduardo Mondlane University, Maputo, Mozambique; 2 Campus Universitário Principal, 257 Maputo, CP Mozambique; 3 Centre of Expertise in in Gender and Diversity, Vrije Universiteit Brussel (VUB), Pleinlaan 2, 1050 Brussels, Belgium; 4 Department of Philosophy and Ethics, Centre of Expertise in Gender and Diversity, Vrije Universiteit Brussel (VUB), Pleinlaan 2, B-1050 Brussels, Belgium; 5 Department of Obstetrics and Gynecology, Faculty of Medicine and Health Sciences, Ghent University Hospital, Ghent University, De Pintelaan 185, 9000 Ghent, Belgium; 6 Department of Sociology and Centre for Longitudinal and Life Course Studies, Universiteit Antwerpen (Antwerp University), Sint-Jacobstraat 2, BE-2000 Antwerpen, Belgium; 7 Centre for Health Systems Research and Development (CHSR&D), University of the Free State, Bloemfontein, South Africa

**Keywords:** Female and male involvement in family planning, Decision-making, Unwanted pregnancy, Barriers to contraception, Mozambique

## Abstract

**Background:**

By focusing upon family planning counselling services, the Mozambican government has significantly enhanced the general health of female and male clients. However, little is known about the experiences of family planning by female and male adults. This article focuses on knowledge, attitudes and practices regarding contraceptive methods and fertility intentions.

**Methods:**

An in-depth qualitative study of female and male clients was conducted in two settings in Maputo province – Ndlavela and Boane. A total of sixteen in-depth interviews, four informal conversations, and observations were equally divided between both study sites. The analysis followed a constructionist approach. Three steps were considered in the analysis: examining commonalities, differences and relationships.

**Results:**

Although there was a high level of family planning knowledge, there were discrepancies in clients’ everyday practices. Male and female clients are confronted with a variety of expectations concerning fertility intentions and family size, and are under pressure in numerous ways.

Social pressures include traditional expectations and meanings connected to having children, as well as religious factors. Short interaction time between clients and health workers is a problem. Additionally, imposed contraceptive methods, and typically brief conversations about birth control between couples only adds to the burden. Because family planning is largely viewed as a woman’s concern, most clients have never attended counselling sessions with their partners. Attitudes towards responsibility for contraceptive use and risk-taking are strongly gendered.

**Conclusions:**

Female and male clients have differing expectations about contraceptive use and fertility intentions. They participate differently in family planning programs leading to their inconsistent and ambivalent practices as well as vague perceptions of risk-taking. Therefore, policymakers must address the reasons behind ambivalence and inconsistency regarding contraceptives and family planning.

## Background

The involvement of both men and women is considered a key element to the success of family planning programmes. It enhances the use and preference of contraceptive methods suitable for both partners [1–5]. Moreover, inter-spousal/partner communication encourages men to get involved in the decision making about family size and spacing of children. However, the level of male involvement in family planning was found to be very low [6]. Yet, men are most often the primary decision-makers when it comes to actual family planning [7–9].

Recently, family planning programmes have been shifting away from strictly focusing upon women to working with men individually as well as consulting with both partners at the same time [10, 11]. Men are told they also have responsibilities while women are informed they should have equal decision-making rights where reproduction is concerned [8].

In many sub-Saharan countries, including Mozambique, women have little or no control over their own reproductive life. However, they are held responsible for obtaining and using contraceptives [6, 7, 12–14]. Differences in decision-making power and preferences between men and women influence the ability of the latter to implement decisions about family planning including sexual and reproductive (SRH) [1, 15–19]. Moreover, resistance from male partners often leads to the failure of family planning programmes [5, 14, 20]. As emphasised by Mosha et al. [20], there is a deep lack of understanding about what influences the use of family planning and how decision-making takes place within families.

Meanwhile, women who want to limit their fertility are often unable to obtain the contraceptives they need due to various barriers. These include long distances to medical clinics, poor distribution of contraceptives, fear of contraceptive side effects, misconceptions, gender inequality between partners (men are often the primary decision-makers), and health providers’ attitudes [20–23].

For instance, misconceptions about IUDs, such as risk of infection, ectopic pregnancy and infertility have been found to downgrade its acceptance. Reproductive intentions and the adoption of contraceptive methods are also influenced by informal social interactions outside the marital unit. These social exchanges are gendered – men and women have separate social spaces of learning, contacts and networks. This tends to reinforce gender differences in attitudes towards family planning [13, 24, 25].

Factors constraining fertility intentions and contraceptive use vary between urban and rural areas. In urban centres, issues such as cost of living, economic constraints, and women’s involvement in many social roles all contribute to altering the value of having children. Conversely, in rural settings other factors take precedence. Reasons such as higher mortality rate, prestige associated with large families, preference for sons, and the economic value of children to help their parents as farm labour, influence fertility intentions and contraceptive use [13, 26].

In Mozambique, there are considerable differences in contraceptive use between rural and urban citizens. The most recent Demographic Health Survey (DHS) reveals urban and rural contraceptive use of 21.1%, and 7.2%, respectively [27]. The same DHS also captured a wide gap in the type of contraceptives used.

Of married or cohabiting women in urban areas 8.5% used birth control pills. In contrast, only 2.8% used modern contraceptive methods in rural areas. A similarly wide gap was found with injections, the second most preferred method of contraception. In urban areas, injections account for 8.3% but only 3.8% used this contraceptive method in rural areas.

At the national level, 95.5% of women and 99.9% of men aged between 15 and 49 years have knowledge about modern contraception. However, there were differences between women and men with regard to the ideal family size. For women, the average was 5.3 children while men averaged at 6.1 children [27]. Although some progress has been achieved in terms of the level of knowledge about modern contraception, much more is needed in terms of practices and attitudes held by Mozambican women and men.

This article focuses on experiences concerning family planning among adult men and women and in particular on clients’ fertility regulation and intentions. It explores how communication dynamics influence family planning decision-making among women and men in relation to their partners, from a social constructionist perspective [28]. Specifically, we take into account the concept of socialisation and social distribution of knowledge.

Socialisation is understood as a process of learning customs, attitudes and values of a social group, community or culture to conform to norms and roles required for integration into a group or community. Social distribution of knowledge is understood as information derived from people’s practical experience of the world [28]. This knowledge is socially distributed according to 1) what is taken for granted; and 2) systems of relevancies (norms, beliefs and myths).

This means adult men and women may possess dissimilar knowledge about family planning which is resultant of different experiences and position within the family and community. Each man and woman operates according to his or her stock of knowledge and background. This implies that clients do not share the same level of knowledge compared to people they may interact with such as healthcare providers, healers or traditional birth attendants.

This study also seeks to identify the content and pattern of decision-making regarding contraceptive use among female and male clients as well as how women and men perceive this process in the context of their own relationships. To do this, we follow the gender perspective in the context of reproduction suggested by Earle and Letherby [29] and Inhorn [30].

Both authors stress that in order to understand women’s health concerns, policymakers and social scientists must take the social context into account. Within the sphere of reproduction, this approach stresses that male gender norms are widely accepted as factors influencing a range of family planning and reproductive health issues. These include informed use of family planning methods and the male partner’s approval of using contraceptives for birth control and/or condoms for disease prevention.

The relevance of combining both social constructionist and gender frameworks further enables understanding of female and male clients’ lived experiences and expectations. As well, it is important to comprehend that men’s and women’s experiences are shaped and prescribed within a set of socially appropriated norms for individuals in a specific culture context. This perspective can also help in comprehending the position of each partner in terms of manifesting their fertility intentions and family size. Thus, the assumptions are:

1. Women and men construct and discuss family planning according to the social norms of their community and are influenced by their social networks.
2. These networks are structured by gender dynamics in regard to contraceptive use, fertility intention, and spacing and limiting family size.
3. Reproductive decisions reflect entrenched male dominance.
4. Women and men are confronted with different expectations and are under different pressures. These include individual choices, coexistence of western and indigenous family models, traditional expectations, and religious factors.
5. Women and men may be influenced by the proximity of Maputo city where urban life may restrict fertility preferences. For instance, couples in urban/peri-urban areas with few economic resources may be inclined to have fewer children due the cost of living. In rural areas, to the contrary, couples with few economic resources may be inclined to have more children because of the value and meaning of each child.

Therefore, this study might contribute to:

1. Understanding how adult women and men perceive and act upon contraception, fertility intentions and spacing and limiting child birth.
2. Understanding the complexities involved in the triangle of adult women/men, health workers, and policymakers,
3. Recommending changes to family planning programmes to improve their outcomes.

## Methods

### Setting

Two sites – Boane and Ndlavela – where selected in Maputo province, Mozambique. Boane is located in the south-west of Maputo province in a rural area. It encompasses an area of 815 km^2^ with a population of 134.000 [31]. Ndlavela is a neighbourhood on the outskirts of Maputo City. It is located at the Infulene Administrative Post in Maputo province with a population of 57.246 [32].

These study sites were selected on the basis of contraceptive prevalence and geographical location. Both are located in Maputo province which, in 2011, reported the highest use of modern contraception, accounting for 32.8% of married women [27]. As a result of the rural to city exodus, people from all over Mozambique mingle to create a cultural mix in both study sites.

Between April and July 2013, this study conducted in-depth interviews with users aged 25–49 years. In-depth interviews were utilised as a way to understand social life beyond outward appearance and to reveal how female and male clients viewed family planning. As Crouch [33] argues, a small number of cases with less than 20 participants can facilitate the researcher’s close association with respondents and enhance the validity of in-depth inquiry.

Therefore, a total of thirty-two interviews were held – sixteen in Ndlavela and sixteen in Boane. Eight female and eight male respondents were interviewed in each study site. In Boane, interviews were subdivided according to the two selected locales: Boane headquarters and Mahubo.

Four female and four male respondents were interviewed in the Boane headquarters while four male and four female respondents were interviewed in Mahubo. Duration of the in-depth interviews was approximately 1 h, 30 min and guided by semi-structured topics. Saturation of information determined the number of in-depth interviews for this study. Table 1 demonstrates characteristics of the participants interviewed during IDIs.

**Table 1:** Characteristics of the participants (adults of reproductive age 25–49)

IDIs with adults aged 25–49	Ndavela/urban		Boane/rural			
	Ndlavela	Ndlavela	Boane head quarters	Boane head quarters	Mahubo	Mahubo
	Female	Male	Female	Male	Female	Male
Number of participants	8	8	4	4	4	4
Age range	29–44	28–49	29–39	27–49	40–48	37–49
Educational level						
Uneducated	1	0	0	0	1	0
Primary school	3	2	1	1	2	1
Secondary school	3	3	2	2	1	3
Beyond secondary school	1	3	1	1	0	0
Number of children range						
1–3	5	4	2	1	1	2
4–6	2	3	1	2	1	1
7–9	1	1	1	1	1	1
10+	0	0	0	0	1	0
Occupation						
Unemployed	1	2	1	0	1	0
Informal seller	2	1	1	1	1	1
Traditional Healer	1	0	0	0	0	0
Public servant	3	4	0	2	0	2
Farmers	0	0	1	0	2	1
Activist of SRH	0	1	0	0	0	0
Teacher	1	0	1	0	0	0
Security sentinel	0	0	0	1	0	0
Type of contraceptive use	Contraceptive use by female	Contraceptive use reported to be used by their female partner	Contraceptive use by female	Contraceptive use reported to be used by their female partner	Contraceptive use by female	Contraceptive use reported to be used by their female partner
	Pills *n* = 2	Pills *n* = 1	Pills *n* = 1	Pills *n* = 1	Pills *n* = 0	Pills *n* = 1
	Injections *n* = 3	Injections *n* = 2	Injections *n* = 2	Injections *n* = 1	Injections *n* = 1	Injections *n* = 1
	Intra uterine devices *n* = 1	Intra uterine devices *n* = 0	Intra uterine devices *n* = 0	Intra uterine devices *n* = 0	Intra uterine devices *n* = 1	Intra uterine devices *n* = 0
	Condoms *n* = 0	Condoms *n* = 1	Condoms *n* = 0	Condoms *n* = 1	Condoms = 0	Condoms *n* = 0
	Withdrawal *n* = 1	Withdrawal *n* = 1	Withdrawal *n* = 1	Withdrawal *n* = 0	Withdrawal *n* = 1	Withdrawal *n* = 1
	Indigenous *n* = 1	Indigenous *n* = 2	Indigenous *n* = 0	Indigenous *n* = 0	Indigenous = 1	Indigenous *n* = 1
	Sterilization *n* = 0	Do not know *n* = 1	Sterilization *n* = 0	Do not know *n* = 1	Sterilization *n* = 0	Sterilization *n* = 0
		Sterilization *n* = 0		Sterilization *n* = 0		

As the table illustrates, all participants of the study sample are between 25 and 49 years-old. This study is part of a broader research entitled *Unravelling the mosaic discourses, and practices about family planning in two settings of Maputo province, Mozambique –* wherein the entire study design considered all clients of reproductive age (15–49 years-old). For the purpose of this study and based on age (adults 25–49 years-old) sex and class, we carefully selected all female and male adults to be included in the sample.

Thus the study collected information about: 1) practices and norms internalised and followed; 2) sources of information upon which they rely; 3) the relation between clients and health workers; 4) subjects perceived as problematic and non-problematic in their daily routine; 5) perceptions about contraceptive methods, family size and the meaning given to children; and 6) barriers to using family planning services.

A total of eight informal conversations were held – four in Ndlavela and four in Boane. In Boane, the informal conversations were subdivided into two sections. Two were held at the Boane headquarters and two in Mahubo. These informal conversations involved both sexes in Boane and Ndlavela.

Lastly, the study used direct observation at the time of data collection. Our observation focused upon: 1) individual characteristics of adults including gestures and nonverbal behaviour; 2) interaction between nurses and users; 3) actions taking place during counselling services; and 4) program promotion in their communities – in the physical surroundings, such as posters and plaques, etc.

This study also used official documents from the Ministry of Health as a means of investigation sustaining the analysis of the study. It reviewed and coded policy documents – specifically the National Family Planning Policy [34], and the Health Sector Policy of 2014–2019 [35]. Here, code is understood as a word or short phrase symbolically assigning a summative, salient, essence-capturing, and/or evocative attribute for a portion of language-based data.

These documents were useful in understanding how the issue of family planning is framed in Mozambique. The assumption of this study is that there is a specific way of framing family planning problems. The strategy and solution includes counselling services at clinics, as well as community activities. Therefore, this study aims to detail how female and male adults interpret contraception, unwanted pregnancies, family planning size and child spacing.

### Interview guide

This study used semi-structured topics for IDIs. During interviews, the main researcher created an interactive relationship with participants by using photos of contraceptive devices. The researcher also encouraged the asking of questions considered controversial. These included discussing responsibility and decision-making concerning the use of contraceptives, experiences of risk-taking behaviour and the meaning of an unwanted pregnancy.

During the interviews, photos of unlabelled contraceptive devices were shared. The first question was “Have you ever seen or heard about these devices or pills”? The interviews next proceeded with questions about prior knowledge and usage of contraceptives, personal experiences, feelings, and expectations about family planning.

### Ethical considerations

This study was approved by the Mozambican National Bio-ethical Committee for Health. Verbal information was provided to all participants so they could make informed choices. Written consent was obtained from all participants who were able to write. However, for participants unable to write, verbal consent was obtained and required someone else known to the participant who could read and sign in their stead. All participants were apprised of the goals of the study and told they were free to withdraw at any time, at any stage and for any reason.

### Data analysis

Interviews were first transcribed, then translated (from Portuguese and Shangana) to English and then coded. This study created open and axial codes [36]. Open codes consist of highlighting what female and male clients reported as occurring more than once, what was said with intensity, and aspects of behaviour that were taken for granted. Open codes encompassed knowledge, language, behaviour, misconceptions and side effects of contraceptives, and perceptions of unwanted pregnancy.

From axial codes, it was possible to create themes or categories by grouping words or phrases. This comprised labelling of disagreements/conflicts, expectations, motivation for using contraceptives, fertility intentions, the meaning of having children, and perceptions of male involvement in family planning.

With both open and axial codes, this study filtered, highlighted and focused on the salient features of the data recorded for generating categories and themes. From these, this study was finally able to comprehend particular significances. Regarding coding, this procedure first followed the “member checking” process. This consisted of consulting the participants during data analysis as a way of validating the findings.

Data were analysed from December 2014 to April 2015 following a social constructionist approach. Three steps were considered in the analysis: examining commonalities, differences and relationships [36]. A triangulation method [37] was applied to discuss the data and to derive greater assurance from the findings. Additionally, this study cross-referenced information from literature review, in-depth interviews, informal conversations and observation of women and men. It also delved into their involvement with family planning. Additionally, the previously mentioned policy documents were referenced – namely the National Family Planning Policy and the Health Sector Policy of 2014–2019.

## Results

Findings of this study included several themes – knowledge, attitudes and practices about contraception, notions about unwanted pregnancy and fertility intentions. Also discussed were perceived barriers to contraceptive use, the relationship between clients and health workers, as well as misconceptions and fear of contraceptive side effects and perceptions about male involvement in family planning.

These themes offer a plausible explanation of female and male experiences with contraception and family planning. Although the examples given in the text are individual voices, they generally represent the perceptions of the majority of participants. In order to protect the identity of study participants, all names used in quotes are fictitious.

### Family planning knowledge

Most female and male clients have knowledge of at least one modern contraceptive method, awareness of child spacing, limiting family size, and the importance of using maternity clinics in the context of provision of family planning. The most known modern contraceptives are pills, injections, IUDs, and male condoms. They also recounted their understanding of natural methods such as withdrawal, periodic abstinence, calendar-based counting routines, and symptoms-based methods (cervical secretions).

However, contraceptives available in the researched areas included implants (only in Ndlavela), female condoms, female sterilisation and vasectomy. Female and male clients relied on various sources of information such as counselling sessions at health facilities and talk in community circles. As well, some indicated they depended on information they heard/saw on television.

*I have heard about family planning at healthcare facility, at home and through conversation with friends* [Flávia, female, 44 years-old, IDI, Ndlavela].

*I hear [family planning] through TV… There are also plaques/signs, and as I like to read…sometimes I search for it and read* … *Family planning is commonly practice by women…* [Bongane, 30 male, years-old, IDI, Boane headquarters].

For most male participants interviewed, family planning is perceived to be a concern of women. The majority of male participants saw no relevance for both members of a couple to discuss fertility intentions or contraceptive methods to be used. Besides, the male contraceptive option was not viewed as important. This study also found differences concerning the level of knowledge about family planning among either of the sexes at both study sites. As it was stated as follows:

*[smiling]…. Like me as I am a mother of three children, I use family planning [injections] to space my pregnancy. The elder [son] is aged 11, the second is aged six, and the third is two years* [Flávia, female, 44 years-old, IDI, Ndlavela].

*… Oh yes, I have heard about family planning here at community, but I never could understood what family planning is about.* [Castigo, male, 43 years-old, IDI, Mahubo].

Among male clients, it was common to hear comments like “I have heard about family planning, but I do not know exactly what it is about”. However, most female and male clients in Ndlavela possessed more accurate knowledge about modern contraceptives than those in Boane. In this study site, most male clients said they had heard about family planning and/or contraception but were not able to verbalise what they understood about it.

This study also found differences in age and gender regarding perceptions about family planning among male and female clients. Younger male and female clients had more accurate information about family planning when compared with older participants. However, in both study sites, female clients had more precise information than male clients.

*“I use to talk acquiescently about tchifala bheleko [contraceptives] within my friends circle”*[Quissana, female, 35 years-old, Boane]

Most participants reported to be dependent upon the sphere of influence of their friends in obtaining information about *tchifala bheleko* – a local term in Shangana for contraception. ***Tchifala*** means to control, block, or prevent, and ***bheleko*** means birth or fertile; therefore, a direct translation is “birth control”. Through informal networks – typically composed of friends, siblings or close relatives – participants share, learn, and chat about modern contraceptive methods and fertility intentions. Some opinions are stated below:

*We talk about business, how to manage the life… Oops, we cannot have many children, it is necessary to take into account the consequences of the cost of life… we talk about diseases. It is necessary to use condom…* [Zavala, male, 39 years-old, IDI, Ndavela].

*There are some friends who say I should not take injections because women who practice it often get cold… At that moment I got ashamed to say I have been taking injections… I had to say I was using pills… about pills there are not many side effects* [Angelina, female, 41 years-old, IDI, Boane headquarters].

Female and male groups are distinctive not only relating to who are members, but also relating to the key topics of conversation. Most female clients focused on “women’s matters” such as family planning, maternal and child issues, relationship with their partners, sexuality, and women’s responsibilities concerning reproduction.

On the contrary, male clients were more concerned with economic issues, HIV/AIDS, fertility, and sexuality within their friends circle. In some circumstances male clients were interested in discussing family planning. Most reported their concern about not being aware of their female partners’ activities, blaming their partners for concealing contraceptive use, or contesting side effects of contraceptives.

Most clients reported having short conversations about contraceptives with their partners. As well, men discussed birth control with their sons while women did the same with their daughters. Conversation between female clients and their daughters was reported to have changed, especially when compared with the time the mothers grew up. An example from the Kudzi’s story follows:

*Oh, rarely I talk about contraception with my husband…My husband does not know which type of contraceptive…It is difficult to guide my daughters. Children nowadays, they do not tell you anything [concerning the first menstruation]. A long time ago we used to tell our mothers and they guided and taught how to proceed… Our mothers used to indicate an elder girl who had also started to see menstruations in order to help us. The neighbours used to bring gifts, and all [adults] knew about it [ritual]. The rule was to stay inside the house without going outside… Oh! It is hard. Things changed…* [Kudzi, female, 44 years-old, IDI, Mahubo].

### Consistent, inconsistent and ambivalent practices about contraceptives

Our findings show the existence of consistent, inconsistent an ambivalent attitudes and practices regarding contraceptives. By consistent practices we refer to a category of women or men reporting the use some form of contraception every time they had a sex with their partners, unless they were trying to get pregnant. Inconsistent practices means a category of women and men reporting not regularly using some form of contraception (modern or natural/traditional) each time they had sex with their partners, unless they were trying to get pregnant. For most females in this category, if their partners encouraged the use of contraceptives, this greatly helped. However, if partners did not want to collaborate, it drastically reduced the use of any contraceptive method including indigenous methods. By indigenous contraceptives, we refer to contraceptive methods based on traditional local practices – this is typically a combination of herbs, amulets, charms and magical medicine, most often provided by traditional healers.

This study considered ambivalent practices as conflicting attitudes and practices including desires about using any kind of modern contraceptive. Thus this study included respondents who provided inconsistent or conflicting responses when it came to contraceptive use and pregnancy. There were three predominant points of view: 1) for these who said it is absolutely important to use modern contraceptive in order to avoid pregnancy, but they would be very pleased if a pregnancy occurred; 2) for those giving somewhat ambivalent midscale responses such as it was important to avoid pregnancy and would be only a little pleased if a pregnancy were discovered; and 3) for those who were entirely indifferent said they “don’t know” or “wouldn’t care” to both questions.

Participants reporting consistent use of modern contraceptives also had their partners’ approval and involvement in family planning. Conversely, inconsistent and ambivalent users of contraceptives were influenced by possible side effects as well as their partners’ negative attitudes:

*He agrees [with contraceptives]… We decided both about the size of our family without the interference of relatives. He often says he does not want more children… We have two children…* [Angelina, female, 41 years-old, IDI, Boane headquarters].

*I think the suitable contraceptives depend on each woman body… But, injections did not suit well on me and I used pills… However I think the satisfactory contraceptive for me is natural methods* [Julieta, female, 44 years-old, IDI, Ndlavela].

Interviewee*: I told her to stop using contraceptives because of the irregularity of her menstruation. When I have no condom I use that way I explained you [withdrawal]. I know how to tune my partner in a tactic that I can ejaculate into her without impregnate.* [To tune means] *… with my wife, here at home, I do not use condom. When I feel that I am almost to ejaculate, I can stretch my wife’s legs in a manner that slowly I am able to lift without she distinguish that I am out. She feels me on the top of her but she does not open completely her legs whereas I am ejaculate. I withdrawal and ejaculate out, it [sperm] does not prop through upon the vagina* [Ngila, male, 44 years-old, IDI, Mahubo].

Consistent practices occurred among participants who were not particularly interfered with by relatives such as their mother-in-laws. As well, being constrained by economic factors is indicative of consistent contraceptive use. Relatives were mentioned to have considerable decision-making power over contraceptive use and family size. They often provide emotional and financial support, food, housing, education and healthcare. However, inconsistent and ambivalent practices are influenced by perceptions of side-effects and myths surrounding modern contraception.

The use of condoms as a contraceptive choice was viewed as a challenging issue. For instance, after starting to use the pill, Khadidja gave them up because of excessive blood loss which she believed to be a side effect. Like most women interviewed, she had never used female condoms. Once Khadidja decided to stop using contraceptives, she soon found herself pregnant. After giving birth, she decided to change to male condoms – used by her husband. However, she confessed he does not like using condoms:

*He [spouse] says he does not know how to use very well… He often says condoms hurts him….that is he does not have a pleasure. Once, my husband felt sick. He had problems of… [she forgot]… he did not tell me what problem was about. He came at night and talked with me. He said: we have to go to healthcare facility tomorrow morning… He went out to search for diseases. It was an STI.* [Khadidja, female, 29 years-old, IDI, Boane Headquarters].

For most male partners condoms are viewed as inappropriate contraceptive methods for married/partnered couples. Condoms are considered a barrier method, only to be used outside the marriage with a mistress or a casual encounter. As well, the majority of women feel it is the responsibility of their male partner to make the decision to use condoms or not.

When participants were asked about their experiences of not using contraceptives, the very interesting story of Mually came up. At the time of our conversation, Mually said she was still having her menstruation but not taking any modern contraceptive to prevent pregnancy. She believed that if it was possible to prevent pregnancy in the natural way, it would be possible to stop it indefinitely. Her story is as follows:

*I stopped childbearing because I am 43 years-old…*.*Yes, I am done, and now I just stay like that… I did nothing* [to stop childbearing]..*. Even when I was able to get pregnant, I did not need to follow family planning. Everything was naturally and all of a sudden. The same happens now to stop… I just stopped because I did not want to have any more children… Since I was younger, I did not need any tablet. Everything was natural [natural contraception. I feel that now I stopped naturally. Maybe the eggs finished* [Mually, female, 43 years-old, IDI, Ndlavela].

Like other women, Mually believed in “the natural way” and by using such methods, it was possible to temporarily or in definitively prevent pregnancy. Within Mually’s social context, they share the idea that if a couple was not constrained by the cost of life, a “*women has to finish all eggs possible before the menopause phase*”. There is a common belief that God prescribed for all women to have as many children as possible until they enter menopause. In order to justify their practices, most women often speak about the anguish and suffering of women who are infertile but have the desire to have a child or the need to obey their destiny.

### The confluence of indigenous, natural and western contraceptive methods

In both study sites, female and male clients reported having knowledge of modern, natural and indigenous contraceptives. Most female participants described having used both. Reasons for combining several types of contraception were connected with possible side effects of modern contraception, access and effectiveness of these methods. Most female clients believe indigenous and natural contraceptives are the solution when modern birth-control proves to be unsatisfactory.

Furthermore, female clients said they rely on “alternative” contraceptive methods while searching for a suitable modern method**.** What follows is an explanation of how indigenous contraceptives work.

*The indigenous contraceptives follow… You select the first menstruation pad and fill a small or small bottle with the pad and herbs. Whatever is your own decision, or both partners’ consensus. Then you need to hide or put in the ground the bottle or snail in a safe place.* [Julieta, female, 44 years-old, IDI, Ndlavela].

*(…) There is a way to closed [*prevent getting pregnant*], and when a woman grows up and need to have children it is open… Once I asked how it is done [*the ritual of preventing pregnancy*] and she only said it is her mother who knows it… Women are not open to talk about it* [Kossa, male, 38 years-old, IDI, Boane headquarters].

Most women deal with the issue of contraception by sharing with their friends and implicitly trust traditional healers and *massungukate*. *Massungukate* are described as old women who are venerated for their experience and wisdom within the family and community. They advise women in matters related to SRH including marriage. These women are believed to know “women’s secrets” and have knowledge of how to proceed with the appropriate ritual when it concerns family planning.

### Contradictions in following lactational amenorrhea method: the interference of *“a muzhi wa chimbitana”*

Findings reveal contradictions between what was prescribed for the lactational amenorrhea contraceptive method and what was practiced by female clients in their everyday lives. These contradictions are linked to beliefs and norms giving importance to administrating the so called “*muzi wa chimbitana*”. This is an indigenous remedy named by combining two words – muzhi (the remedy) which is boiled in a small *chimbitana* (pot).

During amenorrhea – the absence of menstrual period in a woman of reproductive age – female clients are mindful about the recommendations, advantages and risks associated with this method. However, they are inclined to adhere to social norms, and do what is expected of a mother who has a new born baby. The social norm calls for giving babies a liquid made from the juices of certain plants:

*They [nurses] tell as that there is a way for preventing pregnancy using exclusively breastfeeding… How it comes a baby to live without water and other liquids. Our mothers gave us liquids and nothing happened. A child has to drink the indigenous remedy that protect from diseases and protect from all bad spirits.* [Siphocazi, female, 40 years-old, IDI, Mahubo].

Most clients believe children who drink the magic liquid will grow up protected from maladies, such as epilepsy or psychoses, as well as protection from evil spirits.

Fertility intentions, pregnancy perceptions and family size are the next topics of discussion.

### Deconstructing the notion of unwanted pregnancy

This study investigated how unwanted pregnancy is interpreted among female and male clients taking part in family planning as well as whom unwanted pregnancy affects the most. Findings revealed the majority of participants perceived unplanned/unwanted pregnancy differently compared to the medical perspective recommended through counselling. As it follows:

*(…) Being married if I get pregnant there is no way to say unwanted pregnant … It is very difficult to get married and not being able to give birth because it was the norm to pay for the wedding* [Ana, female, 38 years-old, IDI, Ndlaveal].

*In our stage, we did not have such kind of pregnancy [*unwanted pregnancy*]. This matters concerns to adolescents. It is unwanted because adolescents need to continue with their studies…* [Siphocazi, female, 40 years-old, IDI, Mahubo].

Unwanted pregnancy was perceived to be exclusively relevant to adolescents and young adults. Most clients commonly said “it happened”, “God sent the child”, “It is a contra part a woman has to give to her husband”, or “It is fate”. On the contrary, if an adolescent gets pregnant, it is perceived as unintended because it will lead to the girl dropping out of school.

### Prestige of having children: Dhawi’s perceptions

To illustrate the prestige associated to having a child, this study used the Dhawi’s story. Dhawi is a 43 year-old woman married in a traditional ceremony termed *lovolo*, in native language. Like most women interviewed, Dhawi believes a woman gains prestige when she is able to give birth. Living in economic difficulty since she was a child, she believes when her own children grow up, they will look after her. Dhawi’s story follows:

(…) *If you arrive at your marriage and have no children, this will not give gladness to you husband. This situation, do not go down well to any man… Children is the most important thing in the world, it is above everything. Imagine a situation that you are sick, and you may need a help. Look to that children [*she pointed out a child*], she is small but she can give you water… makes tea… That is the reasons I hate that things [*contraception*]…You may have children and nobody can help you to bring up with them, but soon or later that children will help you, they will take care of you…* [Dhawi, female, 43 years-old, IDI, Boane headquarters]*.*

Having as many children as a woman can possibly bear was seen as a guarantee of future subsistence, survival and social integration in old age. Most participants reported that having children it is a way to strengthen the identity of motherhood and fatherhood and is very important to kin and social networks.

*“… Who will take care of me”?* Tekwasse’s fertility intentions

An example of fertility intentions and contraceptive use among female clients is well illustrated by Tekwasse, a woman who bore six children. Tekwasse is reported to have had never used modern contraceptives, using only natural methods. And because her husband was away for long periods working in South Africa, her method of birth control was simple abstinence. Although she had four living children at the time of our conversation, her ambition was to have more. Her story follows below:

*For me, four children it is not enough… It is important to take into account the dead and mental illness. Imagine, if I have only two children and they die…who will take care of me? [smile]. If you have only two children and the death take one, and another one is affect by mental illness… what is going to happen? …We live in countryside and there are many difficulties… My mother had twelve children, however she did not had a car for transportation. But we are here! We can help each other. The idea that few children are enough does not fit. Family planning should target 12 to 15 years-old because these group still need to joke… Adults…need children to take care of them in future. I have heard government saying that adults should limit their children. They have to limit after having all children because government is unable to help us when we get old [smile]* Tekwasse, female, and 42 years-old, IDI, Mahubo]*.*

For the majority participants, there is a perception that:

1. having less than four children is considered not enough;
2. the idea of small family size it is not suitable particularly for clients who live in rural areas characterised by lack of infrastructure, supplies and food;
3. children are the guarantee for survival subsistence for parents when they grow old; and
4. family planning should target adolescents, not adults.

### Between indigenous and “imported” family models

The study disclosed uncertainties among male and female clients regarding family models. Clients’ attitudes, preferences and motivations reflect conflicting views between ideal family size and the actual number of children they already have. These conflicting beliefs are explained by socio-cultural norms, the prestige of having children and the cost of living. Clients desire to have more than two or three children because of the prestige associated with large families. Conversely, clients are under the pressure of economic constraints.

Using Ndawane’s example, this study describes the factors which influenced him to change his fertility intention. Ndawane is a 27 year-old man and he has been in a marital union since he was 19. He has three children aged eight, six and three years old. At the time of the interview, Ndawane was unemployed and his wife was a farmworker. In order to support his family he works in small activities in the informal sector and helping his wife at the farm. His desire was to have more children if he and his wife were able to support them. As it follows:

*We feel that life is getting harder. …For instance, I planted maize…the person who will buy from me? How much will sell it? … This means that there is no business, no employment, we are suffering. At school for instance, it is not allowed for children to go without wearing school uniform… At school there are other requirements, and me as father I may not have conditions… because we as father we did not do higher school, and we are underprivileged… When a child gets at grade 7 the books are expensive. …Sometimes, I am flat. That [*is*] the reason I said I am forced to stop with three children. I need to prepare the future of my children… I was willing to much to have more children.* [Ndawane, male, 27 years-old, IDI, Mahubo].

Some clients complain about the lack of wage labour, higher cost of living and dependence upon the informal sector as the primary means of subsistence survival. Being constantly pressured by all these factors dictates the number of children a man/woman can afford. However, this is likely to be far less than the number of children clients would like to have. All these factors contribute to the incompatibility between western – the so called – “imported”; and indigenous – the “local and traditional” family models.

In the next section, perceived barriers to contraceptive use are presented.

### Relationship between clients and health care people

Findings of this study reveal relationships between health workers and family planning clients are not built on mutual trust. Observational findings confirmed interaction between clients and health workers was minimal. Several women said the most interaction time they had with health workers lasted no more than 8 to 10 min. Some clients reported they were ashamed to initiate conversation or raise questions to health workers because they were allotted such a short time per visit:

*At the time I arrive [*at the healthcare facility*] I follow the queue. When is my time to pass I give the ID registration card. She [*the nurse*] asks you if it is injection or pills, and I answer it is injection. Then, I show my bum and she gives me the injection… Oops, they [*the nurses*] do not like to talk. But, if you raise questions they answer. If not, there is no conversation… They just administer the injection and say you can go out* [Nhambisse, female, 37 years-old, IDI, Ndlavela]*.*

Although conversations with health workers were considered momentary and limiting for clients to express their concerns, it was the time spent/wasted in the waiting room that discouraged many female clients from using family planning services. However, male clients had little to say about counselling procedures. This was because generally, men were absent from consultations and presentations at the healthcare centre. Most male clients did not often look into family planning, or even escort their partners to counselling sessions.

*I cannot explain deeply what happens [*at the time of consultation*] because rarely I go to healthcare centre. I go there if I have got flu or another illness, for instance a fever… But, she [*his wife*] always goes there, because we have a baby* [Felizardo, male, 40 years-old, IDI, Ndlavela].

### Fear of contraceptive side effects and myths about contraceptives

Imaginary or real, misconceptions about side effects of modern contraceptives contributed to their lack of use. Contraceptive methods such as the IUD and female/male sterilisation were considered the least acceptable by both men and women. As a result, very few women used IUDs, and not one person opted for female sterilization or vasectomy. This avoidance occurred because of false impressions about both contraceptive methods. As it follows:

*IUD penetrates into woman’s body and hides itself. Then, it attracts diseases… If a woman does not menstruate automatically she is not a woman. Everyone will interpret as menopause* [Chimbutane, male, 39 years-old, Ndlavela].

*I heard outside… a woman who submitted to sterilisation do not get cold, but she does not have desire for sex. Being submitted to sterilization… when you have sexual intercourse you do not feel nothing … you do not satisfy your husband* [Ancha, female, 41 years-old, IDI, Boane headquarters].

Sterilisation and the IUD are the most rejected contraceptive methods. Rather than actual side effects, most female clients were worried about their male partners’ opinions of specific contraceptive methods. The IUD is often rejected because women say service providers have to insert it into the uterus and they feel uncomfortable about that. Besides, within clients’ sociocultural background, this attitude can be explained by the fact that men and women who submitted to sterilisation were perceived as “eternally infertile” or “possess no femininity or masculinity”.

Female clients tended to be more interested in finding a contraceptive they perceived as more suitable – not necessarily for themselves – but more satisfactory for their partners. There are a variety of arguments: “A woman gets cold”. “When a woman conceals she is using contraception from her spouse, she does it because she has a lover”.

This is particularly associated with the use of IUDs. “It causes blood loss” which is often associated with pills and IUDs. And, “It causes infertility and failure of the eggs”. This last comment is most often applied to people who have opted for female sterilisation or vasectomy in males.

### Men accompanying their partner through counselling practices

Most clients had varying perceptions of the involvement of male partners in counselling sessions. Male clients said that when they see a man escorting his wife/partner to the health centre, he is “labelled as a man who is dominated by his wife”. Other comments included: “it is women’s matter, and it is better that the man let her go alone”; “there is no space for men in the consultation room”; and “it is an obstruction of female matters”. Conversely, several female clients said they were “afraid of being subject to teasing or rumours”, “man often says [he] is not available”, and “there are resistance from the male partners”. This is demonstrated below:

*Women when they see a man in counselling practices, they start to laugh at him…. That is why men do not accept when a woman says lets practice this or those preventive methods. When a man escorts his partner to healthcare facility you hear rumours that you feel bad… Here there an idea that it is only a women concern* [Chitsau, female, 30 years-old, IDI, Ndavela].

*I heard some men saying they went to healthcare facility. I do not know what they did there. They said only they escort their partners. With me, I have never accepted to go to healthcare facility* [Machava, male, 41 years-old, IDI, Boane headquarters].

There were discrepancies between what female and male clients reported having heard in counselling sessions. This study found contradictions between what they learned and what they actually do in practice. A couple going together to counselling a session is not a common practice and contradicts the expectations of gender roles. Moreover, most male clients admitted they paid little attention to information their female partners obtained at counselling sessions, particularly when it concerned family size and contraceptive use.

## Discussion

This study has attempted to shed light on a rarely discussed issue – female and male experiences dealing with family planning. Listening to what clients said relating their personal stories on their own terms seems to be of vital importance to both the academic and policy-making realms.

Firstly, in Ndlavela, there was a stronger trend toward pursuing recommendations from counselling services by female and male clients than in Boane. These differences can be associated with socio-cultural background as well as geographical location. Boane is located in rural Mozambique. Here, the average fertility rate is 6.6 children per woman and the use of modern contraception is 7% [27]. Its inhabitants are more deeply influenced by traditional, social and religious norms [38], and the marital age for girls is 12 [31].

This study found men and women in both study sites possessed a higher level of knowledge/awareness about modern contraceptives. However, there were glaring discrepancies between what clients know and what they actually do. Gender and age differences affected family planning to a significant degree if we consider both study participants and to the participants’ views about young people. Almost all participants between 27 and 35 years-old were more likely to consistently follow any form of contraception or more inclined to collaborate with their partners rather than those who are situated between the age-range of 36 to 49 years-old. However, within the entire male category there was an implicit perception that contraception should target adolescents and young adults. This way of thinking is associated with social and economic consequences of early parenthood rather than considering health consequences of early pregnancies. Meanwhile it is important to consider the differences between rural and urban areas in terms of perception and practices regarding contraception.

These findings are in line with other studies indicating men have limited interest as well as lower level of knowledge about family planning [14, 20, 39, 40]. In this study and others [41, 42], social networks are important learning sources of family planning since they provide various forms of social support such as childcare, housing, money, advice and emotional support.

However, it is important to note these social networks are gendered, and continue to reflect the dominant gender ideology and hierarchy of their culture [13, 24]. On the other hand, clients acknowledged their relationship with health workers was transitory. This may explain the reason why birth control continues to be an unmet societal goal [43–45].

Secondly, neither male nor female clients were interested in vasectomy and female sterilisation. Rejection of this permanent form of contraception is directly related to cultural norms based on fertility. Sterilisation is interpreted as losing the male or female essence within the cultural setting.

Thus, findings of this study reaffirm the conclusions of prior researchers revealing misconstructions and traditional pro-natal beliefs by clients as well as health workers [46–51]. As argued by Adongo et al. [52], vasectomy is often perceived as an act against God. It is considered a form of castration making men feel weak and incapable satisfying their wives sexually.

Indigenous contraceptives have been used at least once by most female clients. The prevalence of indigenous practices, was likewise found in Agadjanian’s study [53] illustrating the complexity and ambiguity of the notions of tradition – indigenous contraceptives; and innovation – modern contraceptives.

The ambiguity between modern and indigenous methods may lead to a definition of what is considered problematic and non-problematic. That is, what is to be accepted, refused, and negotiated in terms of knowledge, behaviour and practices regarding family planning. As Berger and Luckmann [28] accentuate, knowledge is socially distributed. This means individuals involved in family planning initiatives may act in accordance with their culture, individual experiences, gender, age, and class.

Thirdly, pregnancy is only considered unwanted when it is not socially-sanctioned. If it is revealed a woman has used her sexuality in a way deemed culturally-inappropriate, any resulting pregnancy will only then be considered unwanted. These findings are consistent with Izugbara et al. [54] highlighting that pregnancies were portrayed as unwanted when they occurred in particular contexts:

1. It did not reinforce womanhood and a woman as a wife.
2. It was incongruent with traditional beliefs about proper procreation.
3. It revealed the women used her sexuality in culturally unacceptable ways.

Fourthly, conflicting views concerning the number of children a couple desires to have are explained by social norms, such as the prestige of having children and the cost of living. In the urban environment, a large number of children become a financial burden carried by most families.

This is because, in an urban environment, both partners are exposed to numerous pressures as well as the high cost of living. The identity of mother in urban centres has less power/status compared to women living in rural settings and even less so in greater Maputo city. As Agadjanian’s highlights: in “cement city”, the identity of a woman is considered more valuable when mothers also work outside the family home [55]. Taking up residency close to the Maputo city may expose clients to the clash between western and indigenous family models.

Changes in reproductive norms are also linked to the dynamics of marriage and gender relations. Specifically in rural settings, the payment of *lobolo* – bridewealth – is part of individual and collective identity. It connects the living and ideal in a cultural network of interpretations of the world, within a set of constantly changing traditions [55–57].

In the scope of reproduction, *lobolo* is seen as a means to complete a marriage – the man must pay for the woman and the woman must give a birth as compensation for payment paid to her family. As a result, a women’s status is largely determined by her marital status and ability to give birth. Moreover, a child generates a sense of pride not only for helping to preserve the kin, but also maintains the couple’s identities of being feminine and masculine.

This study’s conclusions confirm prior findings that parenthood is essential in creating and maintaining people’s identities [57, 58]. As stated by Francisco [59], there are three reasons explaining why people have many children: 1) slow demographic transition; 2) most of the population is highly dependent upon a precarious subsistence economy; and 3) the lack of infrastructure, such as healthcare facilities, schools, and other public services.

Lastly, it is important to consider some limitations of this study. At the outset, it must be emphasised this is a small qualitative study conducted using in-depth interviews, observation and informal conversations. Interviews were conducted with only one partner of a couple, rather than both. As well, some of the interviews were held in Shangana.

Aside from being familiar with local beliefs, it is possible the first author may still have omitted some possibly important information that might be useful to a non-native researcher. However, an attempt to balance the bias was accomplished by cross checking field notes and opening discussions with other authors. As much as possible, the team bracketed out participants’ own experiences; dialogue and negotiations with participants, assuming the principle of interpretations are temporal, located and always open for reinterpretation.

Lastly, one study setting is located in peri-urban centre while the other is rural. However, both are located very close to Maputo city. Dynamics of urban/city living, such as tight economic circumstances, may be the primary argument for fertility regulation and thus influenced attitudes and behaviours of the participants. Therefore, these findings cannot be generalised.

## Conclusions

This study reveals a plurality of notions about family planning as well as a variety of expectations and conflicting views about ideal family size. There were discrepancies between what clients know and what they actually do. Most clients seemed ambivalent and their use of modern contraception was inconsistent.

The meaning of having a child and family size is the result of a combination of socio-cultural factors: background, poverty, individual and community choices, and gender norms. Clients must ensure their own social assistance through the practice of having many children. Having as many children as possible, guarantees the future of subsistence, survival and social integration in old age.

Their cultural backgrounds and social norms include the expectation their children are duty bound to help their parents when they are old. Having a large number of children is seen as an eventual solution to their current “hard life” condition. Thus, the value of children and the prestige associated with it and expectations of wedlock play a greater role in contraceptive use by limiting its utilisation.

Therefore, these social expectations explain inconsistent and ambivalent practices regarding modern contraceptives. If a married woman does not have a child, she is perceived as an embarrassment to both herself and her husband. Children are also seen as a sign of affirming the symbolic power of being able to bear a child. Children not only provide a sense of pride in preserving kin, but are also significant in maintaining the parents’ identities of femininity and masculinity. These gender identities are an amalgam of social norms and community recognition. It is also a means to strengthen the identity of motherhood and fatherhood and this is very important to kin and social networks.

Finally, results from this study may contain relevant policy implications, by taking into account the need to design program strategies, such as mixed group education sessions for men and women. New and effective strategies need to be developed to involve men in counselling sessions in order to internalise the concept of shared decision-making within a couple.

As well, there is a strong need to provide accurate information about the IUD, female sterilisation and vasectomy. An accelerated fertility decline is urgent to reduce child mortality and morbidity; improve policies and institutions for domestic savings and investments.

In conclusion, it is recommended female education should be increased in regard to gender equity, and social norms concerning fertility must be addressed. In addition, facilities focusing only on men must be provided. This could be accomplished through the creation of special areas or clinics. This separation would help men feel more comfortable to express their views and also foster their interest in family planning information.

## Abbreviations

HIV/AIDS: Human Immunodeficiency Virus/Acquired immunodeficiency Syndrome
IDIs: In-depth interviews
SRH: Sexual and Reproductive Health

## References

[1] Tilahun T, Coene G, Temmerman M (2014). Spousal discordance on fertility preference and its effect on contraceptive practice among married couples in Jimma zone, Ethiopia. Reprod Health.

[2] Kassa M, Abajobir AA, Gedefaw M (2014). Level of male involvement and associated factors in family planning services utilization among married men in Debremarkos town, Northwest Ethiopia. BMC Int Health Human Rights.

[3] Hartman M, Gilles K, Shattuck D (2012). Challenges in couple communication as a result of male-involvement family planning interventions. J Health Commun.

[4] Chuwa M (2012). Male involvement in family planning practice. Afr J Midw Women’s Health.

[5] Lassee A, Becker S (1997). Husband-wife communication about family planning and contraceptive use in Kenya. Int Fam Plann Persp.

[6] Adelekan A, Omoregie P, Edoni E. Male involvment in family planning: Challenges and way forward. Int J Pop Res. 2014. http://dx.doi.org/10.1155/2014/416457.

[7] Mboane R, Bhatta MP (2015). Influence of husband’s healthcare decision making role on a woman’s intention to use contraceptives among Mozambican women. Repr Health.

[8] Akindele RA, Adebimpe WO (2013). Encouraging male involvement in sexual and reproductive health: Family planning service providers’ perspectives. Int J Repr Contrac Obstc Gyn.

[9] Ijadunola MY, Abiona TC, Ijadunola KT (2010). Male involvement in family planning decisions making in Ile-Ife, Osun state, Nigeria. Afr J Repr Health.

[10] Berhane A, Biadgilign S, Amberbir A (2011). Men’s knowledge and spousal communication about modern family planning methods in Ethiopia. Afr J Repr Health.

[11] United Nations (1995). Men’s and women’s contraceptive practices. Pop Newsletter.

[12] Aransiola JO, Akinyemi AI, Fatusi AO (2014). Women’s perceptions and reflections of male partners and couple dynamics in family planning adoption in selected urban slums in Nigeria: A qualitative exploration. BMC Pub Health.

[13] Agadjanian V (2005). Fraught with ambivalence: Reproductive intentions and contraceptive choices in a sub-Saharan fertility transition. Pop Res Pol Review.

[14] Jayalakshmi MS, Ambwani K, Prabhakar PK (2002). A study of male involvement in family planning. Health and Population-Perspectives and Issues.

[15] Stern E, Cooper D, Gibbs A (2015). Gender differences in South African men and women access to and evaluation of informal services of sexual and reproductive health information. Sex Ed: Sexuality, Soc Lear.

[16] Isichei C, Brown P, Isichei M (2015). HIV prevalence and associated risk factors among rural pregnant women in north central Nigeria. American Journal of Health Research.

[17] Garg S, Singh R (2014). Need for integration of gender equity in family planning services. Indian J Med Res.

[18] Reid A, Nalwaldda G, Ntozi J (2014). Barriers to male involvement in contraceptive uptake and reproductive health services: A qualitative study of men and women’s perceptions in two rural districts in Uganda. Repr Health.

[19] Vouking MZ, Evina CD, Tadenfok CN (2014). Male involvement in family planning decision making in sub-Saharan Africa – what the evidence supports. Pan African Medical Journal.

[20] Mosha I, Ruben R, Kakoko D (2013). Family planning decisions, perceptions and gender dynamics among couples in Mwanza, Tanzania: A qualitative study. BMC Public Health.

[21] Ezeah P, Anchonwa C (2015). Gender inequality in reproductive health services and sustainable development in Nigeria: A theoretical analysis. Int J Sociology and Anthropology.

[22] Kabagenyi A, Ndugga P, Wandera SO (2014). Modern contraceptive use among sexually active men in Uganda: Does discussion with a health worker matter?. BMC Public Health.

[23] Littlejohn K (2012). Hormonal contraceptive use and discontinuation because of dissatisfaction: Differences by race and education. Demography.

[24] Khadivzadeh T, Roudsari RL, Bahrami M (2013). The influence of social network on couples’ intention to have the first child. Iran J Reproductive Med.

[25] Bongaarts J, Watkins SC (1996). Social interactions and contemporary fertility transitions. Popul Dev Rev.

[26] Dadoo FN, Tempenis M (2002). Gender, power and reproduction: Rural–urban differences in the relationship between fertility goals and contraceptive use in Kenya. Rural Sociol.

[27] INE and MISAU (Instituto Nacional de Estatistica e Ministério da Saúde). Moçambique, inquérito demográfico e de saúde 2011. Maputo, Moçambique: INE and MISAU; 2013.

[28] Berger P, Luckmann T (1966). The social construction of reality: A treatise in the Sociology of knowledge.

[29] Earle S, Letherby G (2003). Gender, identities & reproduction: Social perspectives.

[30] Inhorn MC (2009). Reproductive disruptions: Gender, technology, and biopolitics in the new millennium.

[31] MAE (Ministério da Administração Estatal) (2012). Perfil do distrito de Boane.

[32] CMCM - Conselho Municipal da Cidade de Matola (2013). Lista da densidade. Posto Administrativo Municipal do Infulene.

[33] Crouch M, McKennzie H (2006). The logic of small samples in interview-based qualitative research. Soc Sci Info.

[34] MISAU (Ministério da Saúde) (2010). Estratégia do planeamento familiar.

[35] MISAU. Plano estratégico do sector saúde: 2014–2019, MISAU 2014, Maputo-Moçambique: MISAU; 2014.

[36] Gibson W, Brown A (2009). Working with qualitative data.

[37] Bryman A (2004). Social research methods.

[38] Agadjanian V (2013). Religious denomination, religious involvement, and modern contraceptive use in Southern Mozambique. Stud Fam Plan.

[39] Glasier A (2010). Acceptability of contraception for men: A review. Contraception.

[40] Char A, Saavala M, Kulmala T (2009). Male perceptions on female sterilization: A community-based study in rural central India. Int Persp Sex Repr Health.

[41] Buber I, Fliegenschnee K. Are you ready for a child? Methodological triangulation on fertility intentions in Austria, Working Papers 3/2011. Vienna, Institute of Demography; 2011.

[42] Kleim S, Klarner A, Bernardi L (2009). Fertility-relevant social networks: Composition, structure, and meaning of personal relationships for fertility intentions. Max Planck Institute for Demographic Research.

[43] Kamhawi S, Underwood C, Murad H (2013). Client-centered counselling improves client satisfaction with family planning visits: Evidence from Irbid, Jordan. Global Health: Sci Pract.

[44] Agha S, Do M (2009). The quality of family planning services and client satisfaction in the public and private sectors in Kenya. Int J Qual Health Care.

[45] Bruce J (1990). Fundamental elements of the quality of care: A simple framework. Stud Fam Plan.

[46] Sakara A, Namoog MY, Badu-Nyarko SK (2015). Misconceptions and rumours about family planning among Moslem males in Tamale metropolis, Ghana. Glob J Interd Soc Sciences.

[47] Ochako R, Mbondo M, Aloo S (2015). Barriers to modern contraceptive methods uptake among young women in Kenya: A qualitative study. BMC Public Health.

[48] Ankomah A, Anyanti J, Adebayo S (2013). Barriers to contraceptive use among married young adults in Nigeria: A qualitative study. Int J Tropical Disease Health.

[49] Brunie A, Tolley EE, Ngabo F (2013). Getting to 70%: Barriers to modern contraceptive use for women in Rwanda. Int J Gynaecol Obstet.

[50] Polis CB, Zabin LS (2012). Missed contraception or misconceptions: Perceived infertility among unmarried young adults in the United States. Persp Sex Reprod Health.

[51] Diamond-Smith N, Campbell M, Madan S (2012). Misinformation and fear of side-effects of family planning. Cult Health Sex.

[52] Adongo PB, Tapsoba P, Phillips JF (2014). “If you do vasectomy and come back here weak, I will divorce you”: A qualitative study of community perceptions about vasectomy in Southern Ghana. BMC Int Health Human Rights.

[53] Agadjanian V (1999). Women’s choice between indigenous and western contraception in urban Mozambique. Women health.

[54] Izugbara CO, Ochako R, Izugbara C. Gender scripts and unwanted pregnancy among urban Kenyan women. Cult, Health & Sex. 2011;(13:9):1031–45. 10.1080/13691058.2011.598947 21777108

[55] Agadjanian V (2010). Economic security, informal resources, and women’s reproductive choices in urban Mozambique. Soc Biol.

[56] Bagnol B (2008). Lovolo e espíritos no sul de Moçambique. Análise Social.

[57] Granjo P (2005). Lobolo em Maputo: Um velho ideoma para as novas vivências conjugais.

[58] McQuillan J, Greil AL, Shreffler KM, et al. The importance of motherhood and fertility intentions among US women. Sociol Perspect. 2014:1–16.

[59] Franciso A. Protecção Social no contexto da transição demográfica moçambicana. Cad IES. 2011; 11.

